# The relationship between self‐forgiveness and psychological wellbeing in prison inmates: The mediating role of mindfulness

**DOI:** 10.1002/cbm.2260

**Published:** 2022-09-02

**Authors:** Giorgia F. Paleari, Francesca Danioni, Sara Pelucchi, Maria Rita Lombrano, Daniel Lumera, Camillo Regalia

**Affiliations:** ^1^ Department of Human and Social Sciences University of Bergamo Bergamo Italy; ^2^ Family Studies and Research University Centre Catholic University of Milan Milano Italy; ^3^ Department of Psychology Catholic University of Milan Milano Italy; ^4^ My Life Design ONLUS Milano Italy

**Keywords:** mediation, mindfulness, prison inmates, psychological wellbeing, self‐forgiveness

## Abstract

**Background:**

Previous research with general population samples has consistently shown that forgiveness and mindfulness facilitate coping with distressing experiences and significantly promote mental health. No study, however, has examined their unique contribution to prisoners' psychological wellbeing nor has considered the different forms of self‐forgiveness among prisoners.

**Aims:**

Our aim was to investigate the role of mindfulness in mediating any association between prisoners' self‐forgiveness and psychological wellbeing and to test whether any such links are moderated by years spent in prison. In this study self‐forgiveness was conceptualised as a multidimensional construct, including presence of genuine self‐forgiveness, absence of pseudo self‐forgiveness and/or absence of self‐punitiveness.

**Methods:**

Participants were recruited from a prison in Northern Italy. Consenting men were asked to complete an anonymous self‐report questionnaire with only a researcher present.

**Results:**

104 male prisoners (mean age 46.63 years, SD 11.38) took part. Findings were that self‐punitiveness was inversely related to well‐being, with mindfulness mediating this relationship, this while controlling for the other dimensions of self‐forgiveness and the perceived severity of the crime committed. Contrary to expectation, we found no direct relationship between genuine self‐forgiveness and well‐being, but the moderated mediation models showed that genuine self‐forgiveness was positively associated with mindfulness and, through this, had an indirect association with wellbeing, significant only for prisoners who had already spent several years in prison.

**Conclusions:**

Our findings confirm that self‐forgiveness is a complex construct, worthy of further investigation among offenders. They suggest that forgiveness interventions for prisoners should include modules aimed at primarily reducing self‐punitive attitudes. Promotion of genuine self‐forgiveness should be tried only with awareness that this is likely to take a very long time. In such circumstances, interventions may promote energy to be invested in mindful processes with a consequent improvement in psychological wellbeing.

## 1 INTRODUCTION

Prisoners report poor psychological wellbeing and mental health (Fazel & Seewald, 2012), showing higher prevalence of psychiatric and stress‐related disorders when compared to the general population (Macciò et al., 2015). Adaptive coping strategies are positively related to prisoners' psychological wellbeing and are more protective in this respect than prison‐related variables, such as length of sentence or time spent in prison (Ávila & Sanjuán, 2018; Gullone et al., 2000). Among the strategies facilitating adaptive coping in prison, mindfulness and forgiveness have recently received some attention. A meta‐analysis of the effects of yoga and mindful meditation among prisoners showed that those who took part in these activities showed higher psychological wellbeing scores and better behavioural functioning (Auty et al., 2017). Other works support the possibility of developing interventions for prisoners specifically focused on forgiveness, which also promote psychological wellbeing (e.g., Akhtar & Barlow, 2018; Enright et al., 2016).

Studies outside the prison context show that forgiveness and mindfulness are consistently related to positive health‐related outcomes (e.g., Gismero‐González et al., 2020; Lomas et al., 2019; Massengale et al., 2017). Research in this area, however, has almost invariably considered these two variables separately, showing a positive effect of each of them on psychological wellbeing. One relevant study by Webb et al. (2013) tested the mediating role of mindfulness in the forgiveness‐health outcomes, finding that forgiveness of others, of self, and of situations relates to mental health through its association with mindfulness. This may be because, as suggested by the (un)forgiveness‐energy hypothesis (Worthington et al., 2001), forgiveness allows negative thoughts and feelings related to the offence (like remorse, rumination, and guilt) to be defused, freeing up energy to be invested in mindful processes (e.g., Pelucchi et al., 2013); this in turns promotes health and engagement in self‐care. Consistent with this hypothesis, recent studies have suggested a connection between forgiveness of others and of self on the one hand and mindfulness on the other (e.g., Arslan & Coşkun, 2021; Karremans et al., 2020).

When considering prisoners, whose offences have important implications for their life, it may be particularly relevant to focus on self‐forgiveness, which was found to be more strongly linked to wellbeing than interpersonal forgiveness (e.g., Tenklova & Slezackova, 2016). Previous research with prisoners evidenced the different nature of forgiveness of others and of self (Barbetta, 2002), but very few studies have specifically investigated the link between self‐forgiveness and wellbeing within this population (e.g., Randall & Bishop, 2013). To the best of our knowledge, no research has explored the path from offence‐related self‐forgiveness to psychological wellbeing nor the mediating role of mindfulness among prisoners.

### 1.1 Self‐forgiveness in prisoners

Self‐forgiveness is a proactive, meaning‐focused coping strategy used to overcome stress following a transgression committed towards other people, or the failure to meet an important standard or expectation (e.g., Pelucchi et al., 2017). Indeed, when a person becomes aware that his/her behaviour caused suffering, whether to another person or to him/her self, different painful emotions that may result in self‐punishment and self‐devaluation, such as guilt and shame, are likely to arise (de Vel et al., 2018; Gilbert & Andrews, 1998). Differently, self‐forgiveness may allow a positive change in the offender's attitude towards the self and promote health (Carpenter et al., 2014; Pelucchi et al., 2017; Toussaint et al., 2017).

Woodyatt and Wenzel (2013b) propose a tri‐partite self‐forgiveness concept and distinguish three possible responses to the self that may follow a transgression: genuine self‐forgiveness, pseudo self‐forgiveness, and self‐punitiveness. Genuine self‐forgiveness is a long and gruelling process that starts when the person becomes aware of his/her own culpability and responsibility and experiences coherent emotions (Strelan, 2017), is then able to make progressive changes in value orientation, accepting her/himself with her/his failures, and regaining a positive self‐image as worthy and honourable (e.g., Worthington & Langberg, 2012). Genuine self‐forgiveness is fully reached when the person is able to maintain these changes over time despite doubts and circumstances that recall the offence committed (Worthington, 2006). Previous research has documented the benefits which derive from genuine self‐forgiveness and its positive relation with the offender's wellbeing (e.g., Cornish et al., 2018). Pseudo self‐forgiveness is instead the minimisation of harm, denial of wrongdoing, or victim derogation where there has been interpersonal transgression, releasing the individual from guilt and shame (Woodyatt & Wenzel, 2013b). This may arise especially when the offender perceives him/herself to be ostracised and that others, including the victim, are hostile. It is a form of defensive response that allows maintenance or restoration of positive self‐regard following a transgression, but without improving psychological wellbeing (Costa et al., 2021; Woodyatt & Wenzel, 2013a). Finally, self‐punitiveness is an internalising response to the consequences of an offence committed, where the ongoing guilt and shame for the action strengthen the desire to punish and devaluate the self, with negative consequences for psychological wellbeing (Costa et al., 2021); in this event, although the offender recognises his/her responsibility, this is not associated with efforts to change (Woodyatt & Wenzel, 2013b).

Previous literature on the topic clearly showed that self‐forgiveness dimensions are related among them: specifically, genuine self‐forgiveness tends to correlate negatively with pseudo self‐forgiveness (e.g., Griffin et al., 2018) and positively with self‐punitiveness (e.g., Woodyatt & Wenzel, 2013b), while the relationship between pseudo self‐forgiveness and self‐punitiveness appears to be more inconsistent (e.g., Woodyatt & Wenzel, 2013b). Moreover, self‐perception of having committed a serious offence has been proved to negatively relate to the self‐forgiveness process (Carpenter et al., 2014; Hall & Fincham, 2008). The rare existing evidence on self‐forgiveness among prisoners showed that it is unrelated to interpersonal forgiveness, but positively and negatively related to guilt‐ and shame‐proneness respectively (Barbetta, 2002; Osei‐Tutu et al., 2021). No studies, however, considered the different forms of self‐forgiveness separately.

### 1.2 Mindfulness in prisoners

Mindfulness is defined as “a receptive attention to and awareness of present events and experience” (Brown et al., 2007, p. 212). Indeed, mindfulness helps to adopt a different, non‐judgemental but more conscious attitude towards one's thoughts (Teasdale et al., 1995). It has been conceptualised both as a relatively enduring disposition and as a short‐term state depending upon circumstances. Research has documented a positive relationship between dispositional mindfulness and positive outcomes as wellbeing and resilience (e.g., Jha et al., 2017; Nila et al., 2016), even in highly stressful environments and situations (e.g., Adarves‐Yorno et al., 2020; Götmann & Bechtoldt, 2021; Seear & Vella‐Brodrick, 2013). Specifically, prisoners' dispositional mindfulness has been found to be inversely related to their levels of stress and somatic complaints and to have beneficial effects on psychological wellbeing (Derlic, 2020; Ifeagwazi et al., 2019; Poorebrahim et al., 2022). Evidence on the beneficial effects of state mindfulness is more limited.

### 1.3 The current study

Our aim, therefore, was to investigate whether a mindfulness disposition while in prison mediates any relationship between each type of self‐forgiveness (genuine, pseudo or self‐punitiveness) and psychological wellbeing in a sample of Italian prisoners, controlling for the other two types of self‐forgiveness and for the self‐perceived severity of the crime committed. Our hypotheses were that genuine self‐forgiveness and self‐punitiveness would uniquely relate to psychological wellbeing respectively through the mediation of dispositional mindfulness (*H1a* and *H1b* respectively). No mediated unique relationship between wellbeing and pseudo self‐forgiveness (*H1c*) was expected.

In addition, given that genuine self‐forgiveness is likely to be a long and gruelling process, we assumed any relationship with mindfulness and psychological wellbeing may become stronger over time; in other words, our second hypothesis was that a mediation model between genuine self‐forgiveness and wellbeing may be moderated by years spent in prison (*H2a*), but models relating self‐punitiveness and pseudo self‐forgiveness to wellbeing (*H2b* and *H2c* respectively) would not.

## 2 METHODS

### 2.1 Ethics

The study was fully compliant with the Italian Psychological Association ethical guidelines for research (AIP, 2015).

### 2.2 Participants

Participants were volunteers from a single prison in Italy, innovative for the opportunities that it offers to inmates in terms of training, study, and work activities. They were contacted by the professional educators working in the penitentiary based on their availability (e.g., not involved in other activities) at the moment of data collection and were informed about the main objective of the study, namely studying prisoners' wellbeing. It was explained to them that their participation was free and voluntary and that if they chose to participate, their participation would not affect their sentence or care in any way. After providing informed consent, they were asked to fill in an anonymous paper self‐report questionnaire. The only inclusion criteria were to be male prisoners and fluent in Italian.

### 2.3 Measures

*Sociodemographic information.* Participants were asked questions about their personal characteristics (age, nationality, years spent in Italy) and about their experience of imprisonment (crime committed, years spent in prison).

*Self‐forgiveness for the crime committed.* We used the Differentiated Process Scales of Self‐forgiveness by Woodyatt and Wenzel (2013b) to measure the extent to which participants had forgiven themselves for the crime committed. The scale consists of 19 items that tap three dimensions of self‐forgiveness, namely genuine self‐forgiveness (item example: “I am trying to learn from my wrongdoing”), pseudo self‐forgiveness (item example: “I wasn't the only one to blame for what happened”), and self‐punitiveness (item example: “What I have done is unforgiveable”). Respondents rated on a 6‐point Likert scale the extent to which they agree with each statement (from 1 = completely disagree to 6 = completely agree). Cronbach's *α* in the present sample was 0.75, 0.62, and 0.72 for genuine self‐forgiveness, pseudo self‐forgiveness, and self‐punitiveness, respectively.

*Mindfulness.* We used a short version of the Mindful Attention Awareness Scale, originally developed by Brown and Ryan (2003). It consists of 5 items to which participants responded referring specifically to their life in prison (an item example: “Here in prison, I rush through activities without being really attentive to them”). Respondents reported how often they had each experience on a 6‐point Likert scale, from 1 = almost never to 6 = almost always (*α* = 0.72 in the present sample). Items were reverse scored to allow computation of a total score indicating degree of mindfulness.

*Wellbeing.* We used the short version of the Psychological General Well‐Being Index by Grossi et al. (2006). The scale consists of 6 items and it measures the overall level of the respondents' wellbeing. An item example is: “I was emotionally stable and sure of myself”. Respondents rated the extent to which they have felt in the way described in the past month on 6‐point Likert scale (from 0 = never to 5 = always). The total score was obtained by summing the responses to all six items (*α* = 0.79 in the present sample). The composite score was given a range of 0 (worst possible level of wellbeing) to 110 (best possible level of wellbeing) in order to facilitate comparison with the longer version of the scale across studies (Grossi et al., 2006).

*Perceived severity of the crime committed.* Participants answered on a 6‐point Likert scale (from 1 = not severe to 6 = extremely severe) the following question: “How serious do you consider the offence that got you into prison this time?”.

### 2.4 Procedure

Consenting participants were asked by researchers to complete paper questionnaires and returned them anonymously.

### 2.5 Data analysis

We used PROCESS 3.3 macro (Hayes, 2018) Model 4 to estimate 3 simple mediation models, one for each dimension of self‐forgiveness. In each model, a self‐forgiveness dimension was entered as independent variable (*X*), mindfulness as mediator (*M*) and wellbeing as dependent variable (*Y*), and the two other self‐forgiveness dimensions and the perceived severity of the crime committed were included as covariates.

We then used Model 8 of PROCESS 3.3 macro to test if the years spent in prison (*W*) moderated the relationships in any of the three models tested above. Model 8 estimates any indirect effect of self‐forgiveness (*X*) by the years spent in prison (*W*) on wellbeing (*Y*) through mindfulness (*M*) by first considering the interaction effect of *X* and *W* with *Y*, secondly, the interaction effect of *X* and *W* with *M* and, finally, the main effect of *M* with *Y*. Moderated mediation examines whether the magnitude of a mediation effect depends on the value of a moderator (Muller et al., 2005). The significance of the moderated mediation (i.e. of the difference of the indirect effects across levels of years spent in prison) was tested through the index of moderated implemented in PROCESS (Hayes, 2015). Again, we tested one separate model for each dimension of self‐forgiveness, and the remaining dimensions of self‐forgiveness and the perceived severity of the crime committed were included as covariates. We followed a bootstrap approach based on 5000 bootstrap samples using a 95% bias‐corrected confidence interval (Lau & Cheung, 2012; Preacher & Hayes, 2008; Shrout & Bolger, 2002) to test the significance of indirect and total effects in the mediation models. Confidence intervals that do not contain zero indicate effects that are significant.

## 3 RESULTS

One hundred and thirteen men in the prison agreed to participate, but only 104 fully completed the questionnaire. Their mean age was 46.63 years (SD = 11.38; range 24–75). On average, participants had already spent 9.37 years in prison (SD = 7.94, range 1–42). Fifty‐one participants (49%) had been convicted of homicide, 14 of robbery (13%), 12 of sexual offence (12%), seven of attempted homicide (7%) and the remaining (*n* = 20; 19%) of other offences. Most participants were native Italians (*n* = 81; 78%); the others had been living in Italy, on average, for 17.22 years (SD = 11.53, range 5–50).

In Table 1, we report the descriptive statistics of the variables and the Pearson correlations between them. Respondents showed high levels of genuine self‐forgiveness, followed by self‐punitiveness and pseudo self‐forgiveness. They also showed medium to high levels of mindfulness and wellbeing. Finally, they perceived on average their offence as very severe.

**TABLE 1:** Descriptive statistics and Pearson correlations among variables

Variable	1.	2.	3.	4.	5.	6.	Mean	SD	Range
1. Genuine self‐forgiveness	1						5.12	0.95	1–6
2. Pseudo self‐forgiveness	−0.20	1					2.52	1.05	1–5.50
3. Self‐punitiveness	0.42**	−0.14	1				3.11	1.10	1–6
4. Mindfulness	0.00	−0.20*	−0.27**	1			4.92	0.82	1.80–6
5. Wellbeing	0.00	−0.16	−0.29**	0.56**	1		69.70	19.64	25.67–110
6. Perceived severity of the offence	0.28**	−0.32**	0.30**	−0.02	−0.07	1	4.88	1.44	1–6

*p < 0.05, **p < 0.01.

Self‐punitiveness was negatively correlated with wellbeing, whereas pseudo self‐forgiveness and genuine self‐forgiveness were not. The lack of a significant correlation between genuine self‐forgiveness and wellbeing was unexpected, but might have several explanations, including the fact that bivariate correlations did not control for levels of self‐punitiveness and perceived offence severity, which correlated with genuine self‐forgiveness and well‐being, possibly resulting in a case of inconsistent mediation, that is when mediated and direct effects have opposing signs (MacKinnon et al., 2000); further, the simple test of relationship between genuine self‐forgiveness and wellbeing did not allow for the hypothesised moderating role of years spent in prison. For these reasons, and consistent with recent guidelines for testing mediation (e.g., O'Rourke & MacKinnon, 2018), we estimated simple mediation and moderated mediation models for every forgiveness dimension even if it did not correlate to wellbeing. Self‐punitiveness and pseudo self‐forgiveness, but not genuine self‐forgiveness, were significantly correlated to mindfulness, which in turn correlated with wellbeing. Finally, the finding that perceived offence severity correlated with genuine self‐forgiveness, pseudo self‐forgiveness, and self‐punitiveness as well as genuine self‐forgiveness correlated with self‐punitiveness underlines the importance to control, when estimating mediation models, for offence severity and forgiveness dimensions other than the one entered as independent variable.

**FIGURE 1:**
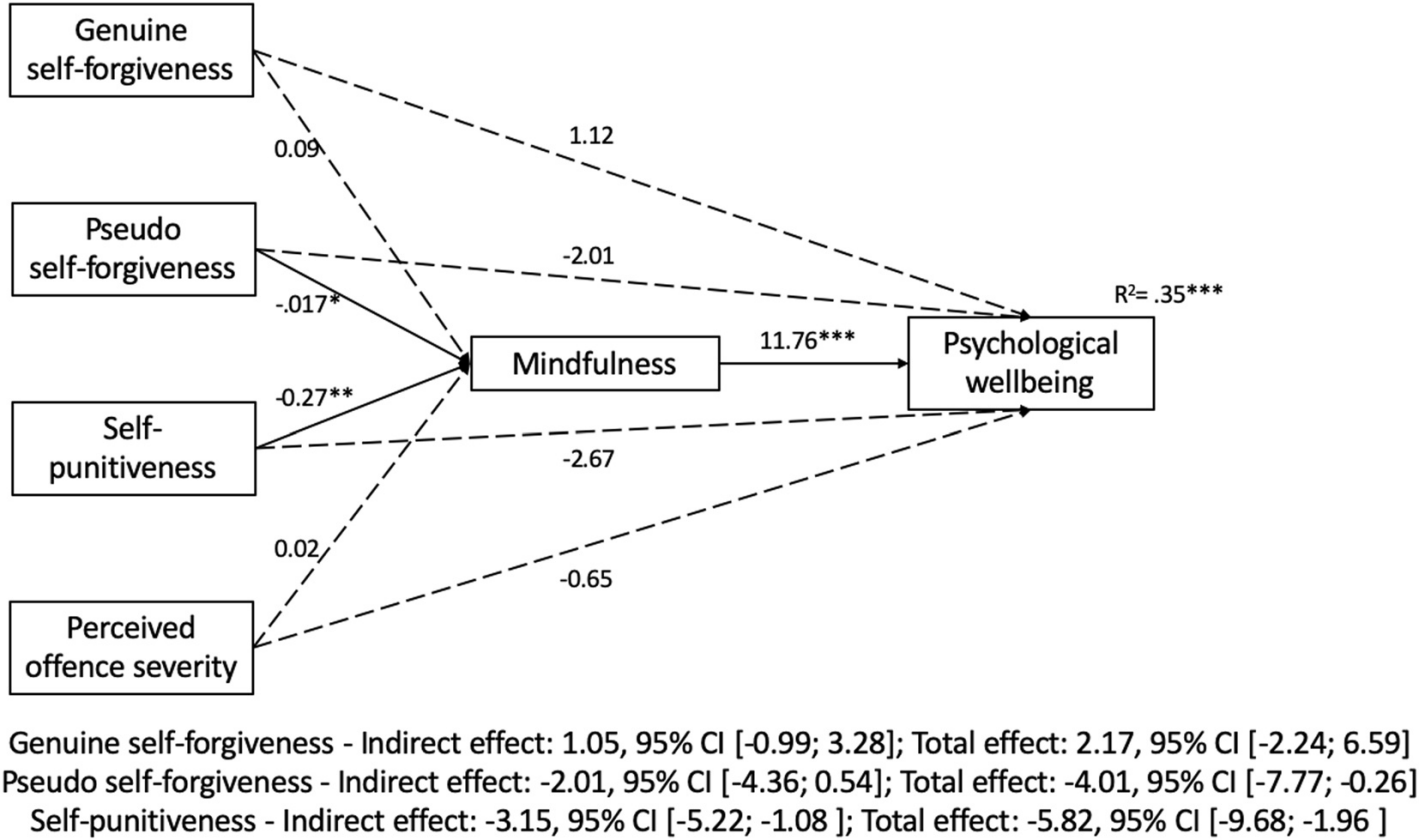
B coefficients for all simple mediation models tested. *p < 0.05, **p < 0.01, ***p < 0.001. CI, confidence intervals

Figure 1 shows the simple mediation analyses, exploring the relationships between each of the three types of self‐forgiveness and psychological well‐being with mindfulness as a possible mediator, while controlling for the other two forgiveness dimensions and for offence severity. These showed that neither genuine nor pseudo self‐forgiveness were related to wellbeing, whether directly or indirectly (contrary to *H1a* and in line with *H1c,* respectively). There was no direct relationship between self‐punitiveness and wellbeing, but an indirect, inverse relationship was found, mediated by mindfulness (consistent with *H1b*). In all models, mindfulness was strongly related with wellbeing.

**FIGURE 2:**
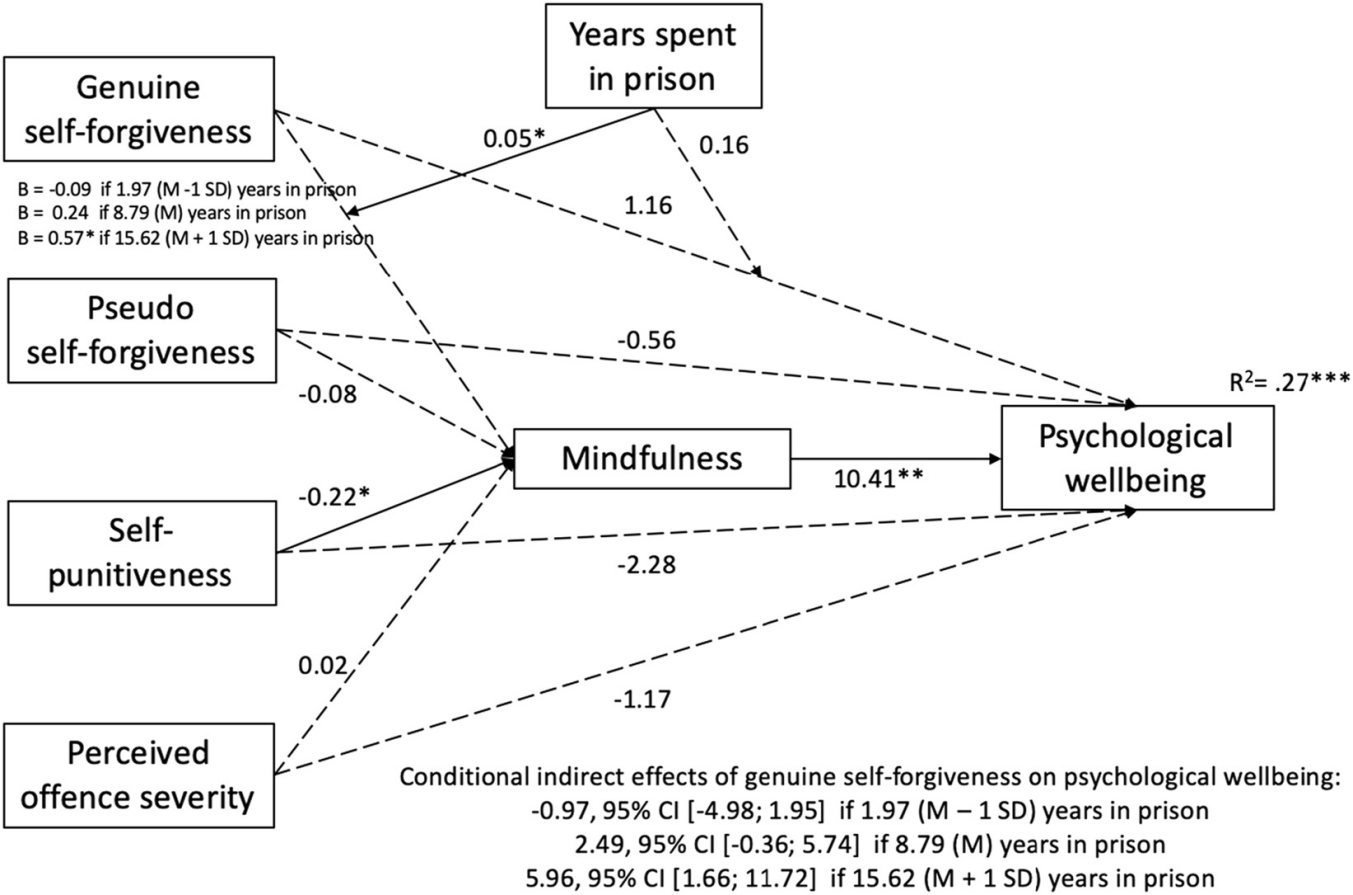
B coefficients for moderated mediation model in which years spent in prison moderated to the links of genuine self‐forgiveness with mindfulness and wellbeing. *p < 0.05, **p < 0.01. CI, confidence intervals

When moderated mediation models were estimated, the index of moderated mediation was statistically significant only for the model in which wellbeing was the dependent variable and genuine self‐forgiveness was entered as independent variable (Index = 0.51, SE = 0.23, 95% CI [0.12, 1.01]), not for the models in which pseudo self‐forgiveness (Index = 0.04, SE = 0.24, 95% CI [−0.58; 0.38]) or self‐punitiveness (Index = 0.41, SE = 0.29, 95% CI [−0.09; 1.04) were entered as independent variable (in line with *H2a*, *H2b* and *H2c*). When controlling for the perceived severity of the offence, for self‐punitiveness and for pseudo self‐forgiveness, the relationship between genuine self‐forgiveness and mindfulness was moderated by the years spent in prison (*B* = 0.05*), whereas the direct relationship between genuine self‐forgiveness and wellbeing was not moderated by years spent in prison when the effect of mindfulness on wellbeing was controlled (*B* = 0.16, *ns*) (see Figure 2).

Conditional effects indicated that genuine self‐forgiveness was significantly and positively related with mindfulness (*B* = 0.57*) only for prisoners who had spent several years in prison (1 SD above the mean); specifically, the Johnson‐Neyman analyses showed that genuine self‐forgiveness and wellbeing are significantly related only when these men had spent more than 10 years in prison (*B* = 0.32*). This also held for the indirect relation between genuine self‐forgiveness and wellbeing, which was significant only when men had spent years in prison one standard deviation above the mean (*B* = 5.96, 95% [1.66; 11.72].

The simple mediation and moderated mediation models were also tested inverting the independent variables (i.e., self‐forgiveness dimensions) and the mediator variable (i.e., mindfulness) in order to verify whether the former might act as mediators of the relation between mindfulness and wellbeing. When mindfulness was entered as independent variable (*X*) and the self‐forgiveness dimensions as simultaneous mediators (*M*) no mediation effect emerged either in the simple mediation nor in the moderated mediation model, thereby refuting this alternative hypothesis.

## 4 DISCUSSION

The aim of our study was to explore the role of mindfulness in any relationship between self‐forgiveness in one or more of its three forms ‐ genuine self‐forgiveness, pseudo self‐forgiveness and self‐punitiveness ‐ and psychological wellbeing in a sample of Italian prisoners. Contrary to evidence from the general population (e.g., Cornish et al., 2018), prisoner's genuine self‐forgiveness was not overall related to wellbeing, neither directly nor through the mediation of mindfulness, but was related to it only among the men who had spent longer in prison (at least 10 years). Indeed, years spent in prison were found to moderate the indirect effects of genuine self‐forgiveness on psychological wellbeing: the positive association of genuine self‐forgiveness with disposition towards mindfulness, which in turn positively related to wellbeing, resulted to become stronger as a function of the years spent in prison. Thus, our results suggest that, for people responsible for very serious offences, genuine self‐forgiveness requires a lot of time and effort not only to be attained (Strelan, 2017), but also to produce its positive effects in terms of freeing up energy to be invested in mindful processes and thereby indirectly promoting wellbeing (e.g., Pelucchi et al., 2013; Worthington et al., 2001). This might happen because, when recently granted, genuine forgiveness and the decision to forgive can be unstable and called into question by circumstances that recall the offence committed. For example, according to the REACH (R = Recall the hurt; E = Empathize with your partner; A = Altruistic gift; C = Commit; H = Hold onto forgiveness) forgiveness model by Worthington (2006), the last step to reach a full genuine forgiveness consists of holding onto forgiveness when doubts occur. Prisoners' genuine self‐forgiveness might have effects delayed over time because both the severity of the offence and the difficulty to make amends and to receive the victim's forgiveness hinder the definitive granting of genuine self‐forgiveness. Previous research has highlighted that self‐forgiveness can be facilitated by the offender's reparative strategies and the victim's forgiveness (Costa et al., 2021; Pelucchi et al., 2015); when the reparation of the harm done and the victim's interpersonal forgiveness are difficult or even impossible to achieve, as it mostly happens to prisoners, genuine self‐forgiveness may require a lot of time to become stable and manifest its positive effects.

Consistent with previous studies on the general population (Costa et al., 2021), prisoners' self‐punitiveness resulted to be uniquely associated with their wellbeing, through the mediation of mindfulness. Specifically, having more punitive attitudes towards themselves was related in prisoners being less prone to be mindful in daily life, which in turn was negatively related to their psychological wellbeing. Therefore, our result suggests that self‐punitiveness may have a negative effect on wellbeing by reducing the mindful disposition, consistent with the (un)forgiveness‐energy hypothesis (Worthington et al., 2001). Also, the result that self‐punitiveness related to mindfulness and psychological wellbeing independently of years spent in prison, whereas genuine self‐forgiveness did not, is to some extent consistent with a substantial body of literature showing that “bad is stronger than good” (Baumeister et al., 2001, p. 362); negative events tend to influence emotion, cognition, and behaviour more strongly than positive ones (Rozin & Royzman, 2001). Similarly, self‐forgiveness (and interpersonal forgiveness) research shows that regardless of the scale used to measure it, the negative dimension of forgiveness (or self‐punitiveness) is a better predictor of wellbeing than the positive dimension (or genuine self‐forgiveness).

Finally, consistent with previous evidence from general population studies (Costa et al., 2021), prisoners' pseudo self‐forgiveness was unrelated, both directly and through the mediation of mindfulness, to wellbeing, even when considering years spent in prison. Indeed, the defensive downplaying of responsibility and wrongdoing might prevent people from working through the transgression, from reflecting on themselves and making those changes needed to improve their wellbeing and avoid pathological outcomes (e.g., Wohl et al., 2017).

Overall, our results suggest that strategies to reduce punitive attitudes towards the self might be more effective in promoting mindfulness and wellbeing among prisoners than the strategies averting pseudo self‐forgiveness or fostering genuine self‐forgiveness. Compared to the general population, self‐harm behaviours are significantly more prevalent among prisoners (Fazel et al., 2016): it seems, therefore, of primary importance that prisoners receive early intervention to counteract self‐punitive attitudes. Interventions fostering genuine self‐forgiveness might have similar positive outcomes but, in the long term, when prisoners have had the time to attain permanent changes in value orientation and self‐image which genuine self‐forgiveness entails. Also, since it resulted more proximally and strongly related to wellbeing than self‐punitiveness and genuine self‐forgiveness, mindfulness is confirmed as an important feature through which to implement interventions to build or protect prisoner's wellbeing (see also Auty et al.’s (2017) meta‐analysis). In summary, decreasing self‐punitiveness and increasing genuine self‐forgiveness and mindfulness appear to be possible strategies inmates may work on to enhance their wellbeing.

### 4.1 Strengths and limitations

This study has some strong points. Unlike most research on the topic, which has conceived self‐forgiveness as a unidimensional construct, the tri‐partite model of self‐forgiveness which informed our study (Woodyatt & Wenzel, 2013b) allowed us to detect the unique direct and indirect relations that each form of self‐forgiveness has with wellbeing, thereby bringing out their peculiarities and the complexity of the self‐forgiveness construct. Also, to the best of our knowledge this is the only study which considers the interplay between self‐forgiveness, mindfulness and wellbeing in a sample of prisoners.

As with all studies, however, ours has some limitations. The cross‐sectional design prevented causal inferences and considerations regarding the directionality of the links among self‐forgiveness dimensions as well as between them and wellbeing via mindfulness. Longitudinal studies are therefore an essential next step towards understanding healthy processing of harmful acts. Also, the sample was one of convenience; participants were selected according to their availability at the moment of data collection and their willingness to take part in the study.

## 5 CONCLUSIONS

In summary, our findings confirm that self‐forgiveness is a complex construct, worthy of further investigation among offenders. There was no correlation between genuine self‐forgiveness and wellbeing among these convicted male prisoners as a whole sample. The relationships between self‐punitive scores and wellbeing and between genuine self‐forgiveness and wellbeing being only among men who had already served longer in prison suggest an important process in which mindfulness has a key role. Longitudinal studies and specific intervention studies might clarify ways of using this line of understanding offenders to promote recovery and longer‐term safety.

Our findings support the relevance of interventions fostering self‐forgiveness and mindfulness among people imprisoned for serious offences. In particular, our findings suggest that forgiveness interventions for prisoners should include modules aimed at primarily reducing self‐punitive attitudes and secondarily promoting genuine self‐forgiveness, while being aware that genuine self‐forgiveness is likely to take a very long time to be fully achieved and have positive outcomes. In such circumstances not only reduced self‐punitiveness, but also increased genuine self‐forgiveness, may promote energy to be invested in mindful processes with a consequent improvement in psychological wellbeing and, perhaps in turn, prosocial behaviours.

## CONFLICTING INTERESTS

The author(s) declared the following potential conflicts of interest with respect to the research, authorship, and/or publication of this article: This research was made possible thanks to the financial support of My Life Design Ente del Terzo Settore–O.D.V. which is the affiliation of two of the co‐authors.

## Data Availability

The datasets generated during and/or analysed during the current study are not publicly available because of local legal and privacy restrictions (Italian Data Protection Code—Legislative Decree No. 196/2003). However, the raw data supporting the conclusions of this manuscript can be made available by the corresponding author to qualified researchers upon request.
